# Role of Polymer Concentration on the Release Rates of Proteins from Single- and Double-Network Hydrogels

**DOI:** 10.3390/ijms242316970

**Published:** 2023-11-30

**Authors:** Daryn Browne, Francesca Briggs, Prashanth Asuri

**Affiliations:** Department of Bioengineering, Santa Clara University, Santa Clara, CA 95053, USA; dbrowne@scu.edu (D.B.); fbriggs@scu.edu (F.B.)

**Keywords:** protein delivery, controlled release, polymer concentration, hydrogel crosslinking density, double-network hydrogels

## Abstract

Controlled delivery of proteins has immense potential for the treatment of various human diseases, but effective strategies for their delivery are required before this potential can be fully realized. Recent research has identified hydrogels as a promising option for the controlled delivery of therapeutic proteins, owing to their ability to respond to diverse chemical and biological stimuli, as well as their customizable properties that allow for desired delivery rates. This study utilized alginate and chitosan as model polymers to investigate the effects of hydrogel properties on protein release rates. The results demonstrated that polymer properties, concentration, and crosslinking density, as well as their responses to pH, can be tailored to regulate protein release rates. The study also revealed that hydrogels may be combined to create double-network hydrogels to provide an additional metric to control protein release rates. Furthermore, the hydrogel scaffolds were also found to preserve the long-term function and structure of encapsulated proteins before their release from the hydrogels. In conclusion, this research demonstrates the significance of integrating porosity and response to stimuli as orthogonal control parameters when designing hydrogel-based scaffolds for therapeutic protein release.

## 1. Introduction

Hydrogels have tremendous potential for applications in regenerative medicine and biomedical engineering, including tissue engineering, wound dressings, drug delivery, and in vitro screening platforms, owing to their low toxicity, excellent biocompatibility, and tunable physical properties [1,2,3,4,5,6,7,8]. Hydrogels have gained special attention for applications related to drug delivery, especially those related to the delivery of therapeutic proteins [9,10,11]. Hydrogels can respond to a variety of chemical and biological stimuli and can be customized to provide desired delivery rates, thus providing a flexible platform for the delivery of proteins. Hydrogels can also increase the stability of encapsulated proteins, as well as protect them from degradation [12,13,14,15,16,17]. Previous studies have demonstrated that the physical properties of drug delivery systems, including the surface area to volume ratio (SVR), can impact drug release rates [18,19,20,21]. SVR is dictated by the architecture of the scaffold, and recent advances in the fabrication of hydrogel-based micro- and nanofibers with pre-determined properties, including scaffold architecture and dimensions, have optimized their applicability as drug delivery systems [22,23,24,25]. Such hydrogel fibers prepared using either electrospinning, extrusion, microfluidic fabrication, or 3D printing have been used to deliver proteins in a variety of contexts, including the delivery of growth factors for wound healing, tissue repair, and regeneration [25,26,27].

**Scheme 1:**
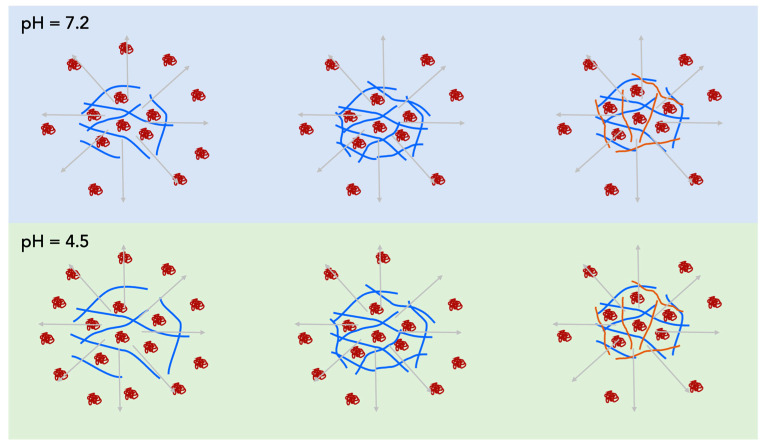
Proposed mechanisms for the proof-of-concept study that demonstrate the impact of innate polymer properties and responses to external stimuli (e.g., pH) to control protein release rates from hydrogel scaffolds.

Other properties that affect protein release rates include the response of hydrogels to various stimuli (e.g., pH, temperature, etc.), as well as the polymer concentration and crosslinking density of the polymeric network, which in turn impacts the porosity of the hydrogel [28,29,30,31,32,33,34]. Researchers have also demonstrated the use of composite hydrogels, such as interpenetrating network and double-network hydrogels that are prepared by combining two different polymers, to develop hydrogel-based platforms with tunable network properties to regulate release rates of therapeutics [35,36,37]. These properties may be combined with SVR as orthogonal design features to optimize drug release rates from hydrogel scaffolds. In this study, we studied the effect of hydrogel concentration and crosslinking density on protein release rates. Others have already demonstrated a direct influence of these properties on protein delivery rates [5,29,38,39]. However, we believe that a more systematic approach to understanding the role of polymer concentration and crosslinking density on release rates, as described in this paper, is necessary before these innate properties are used in combination with SVR to develop optimal hydrogel fiber-based protein delivery platforms (Scheme 1). In this proof-of-study work, we used alginate and chitosan as model polymers to study the roles of polymer and crosslinker concentrations on the release rates of proteins. Our results demonstrate a strong role for polymer concentration and crosslinking density in the protein release rates using single-network and composite hydrogels, as well as in the utility of these scaffolds to preserve the long-term function and structure of the encapsulated proteins before their release.

## 2. Results

### 2.1. Influence of Polymer Concentration on the Release of Proteins

**Figure 1:**
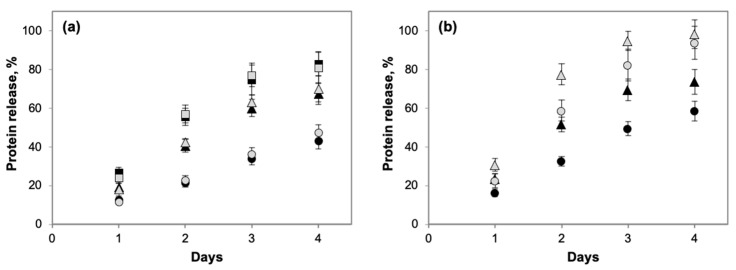
HSA released (%) from hydrogels prepared either using (**a**) 1% (squares), 2% (triangles), and 3% (circles) alginate or (**b**) 0.5% (triangles) and 1% (circles) chitosan at pH 7.2 (black) and pH 4.5 (grey). Data shown are the mean and standard error of triplicate measurements.

We used biopolymers, alginate, and chitosan to study the effect of polymer concentration on the release rates of human serum albumin (HSA), a well-characterized model protein, under physiological conditions (i.e., neutral pH and 37 °C). Both polymers are well-characterized, naturally occurring biopolymers and have been fabricated into fibers using the aforementioned techniques for various biomedical applications [22,24,40,41,42,43,44,45]. Our results clearly demonstrate a negative correlation between polymer concentration and protein release rates for both alginate and chitosan (Figure 1a,b), consistent with previous studies on the effects of polymer concentration on the release rates of various biomolecules, including small molecules, proteins, and nucleotides [5,46,47,48]. Our initial analyses suggest first-order kinetics for the protein release from the hydrogels; however, further experiments with additional time-points must be performed before we may propose and confirm the kinetics of protein release. We also repeated these experiments under low pH conditions to simulate drug delivery to the stomach or the tumor microenvironments [49,50]. Consistent with previous studies, low pH conditions used in this study (pH 4.5) had minimal effect on alginate scaffolds [51] and did not impact the protein release rates from alginate. However, we observed differences in release rates of HSA from chitosan scaffolds at different pH conditions, with increased protein release rates at low pH. These results are in good agreement with previous observations of the effects of pH on the release rates of proteins from chitosan [52,53,54]. Importantly, polymer concentration continued to affect protein release rates from chitosan, even under low pH conditions.

### 2.2. Protein Release Rates from Double-Network Hydrogels

**Figure 2:**
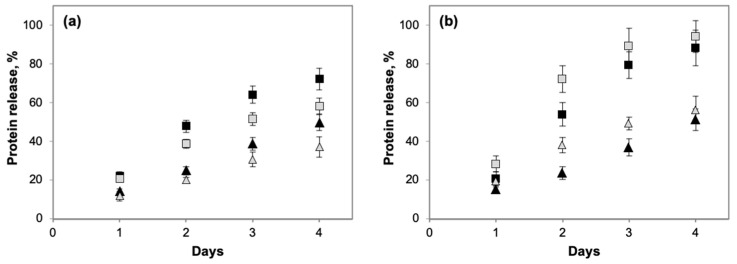
HSA released (%) from double-network hydrogels prepared using 1% alginate|0.5% chitosan (black squares), 1% alginate|1% chitosan (grey squares), 2% alginate|0.5% chitosan (black triangles), or 2% alginate|1% chitosan (grey triangles) at (**a**) pH 7.2 and (**b**) pH 4.5. Data shown are the mean and standard error of triplicate measurements.

Having established the effect of polymer concentration on HSA release rates for single-network hydrogels, we next investigated whether the polymers may be combined to further control the rates of protein release. Previous studies, both conducted in our laboratory and elsewhere [55,56,57,58,59], have demonstrated the utility of developing double-network (DN) hydrogels for a variety of applications in tissue engineering. DN hydrogels demonstrate improved properties relative to their single-network counterparts due to their unique network structure, wherein the different physical properties of the two polymer networks may serve to complement each other [60,61]. Our experiments demonstrated that these observations may be extended to protein delivery applications as well; specifically, Figure 2 demonstrates the ability to control protein release from alginate–chitosan DN hydrogels by modifying the concentrations of either alginate, chitosan, or both. Conveniently, these observations were made for both normal and acidic pH, indicating the utility of DN hydrogels for a range of protein delivery applications. Consistent with the observations made for single-network alginate and chitosan hydrogels, the ability to control the protein release from DN hydrogels using pH was impacted by the concentration of chitosan relative to alginate. For both concentrations of alginate, we observed higher protein release rates for DN hydrogels containing a higher amount of chitosan at low pH conditions. Finally, similar to the single-network hydrogels, our initial analyses (albeit using a limited number of time-points) suggest first-order kinetics for the protein release from the alginate–chitosan DN hydrogels.

### 2.3. Rates of Hydrogel Swelling and Protein Release Are Correlated

**Figure 3:**
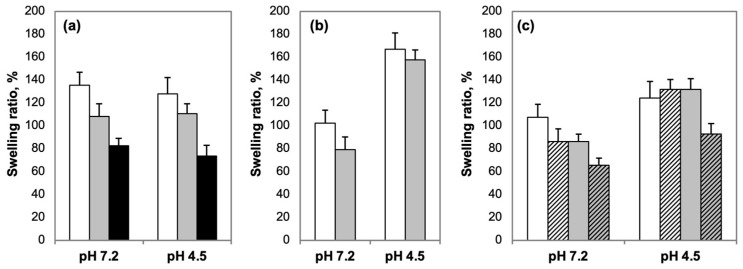
Swelling ratios (%) of hydrogels after 72 h incubation in pH 7.2 or pH 4.5 buffers, prepared using either (**a**) 1% (white bars), 2% (grey bars), and 3% (black bars) alginate; (**b**) 0.5% (white bars) and 1% (grey bars) chitosan; or (**c**) 1% alginate|0.5% chitosan (white bars), 2% alginate|0.5% chitosan (white striped bars), 1% alginate|1% chitosan (grey bars), or 2% alginate|1% chitosan (grey striped bars) at pH 7.2 and pH 4.5. Data shown are the average and standard error of triplicate measurements.

Previous studies have suggested that a variety of factors, including polymer concentration and the addition of fillers (e.g., nanoparticles and polymers), may contribute to the extent of crosslinking in the hydrogel network and thereby affect their swelling rates [31,57,62,63]. While DN hydrogels differ from simple filler-reinforced hydrogels, both in terms of structure as well as properties, we hypothesize that DN hydrogels may similarly benefit from the combination of two hydrogels with different properties. We compared the swellability of both single- and double-network hydrogels prepared using different concentrations of alginate and chitosan at normal and acidic pH (Figure 3). These experiments are relevant, as previous studies have demonstrated a strong correlation between swelling and drug release rates [64,65]. Not surprisingly, these data indicate that polymer concentrations affected the swelling of both alginate and chitosan hydrogels. These experiments also indicated higher swelling rates of chitosan at low pH, but not for alginate (Figure 3a,b). Similar observations, i.e., a negative correlation between polymer concentration and swelling rates, were made for alginate–chitosan DN hydrogels (Figure 3c). More importantly, the swelling experiments revealed the significance of the alginate:chitosan ratio for the DN hydrogels in their swelling under low pH conditions. At low pH, DN hydrogels with higher concentrations of chitosan showed a higher swelling capacity. Taken together, these experiments strongly suggest a strong correlation and even a causation relation between DN hydrogel swelling and protein release rates from DN hydrogels.

### 2.4. Proteins Released from the Hydrogels Retain Activity and Structure

**Figure 4:**
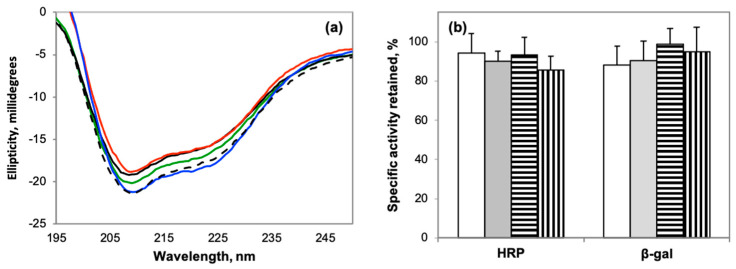
(**a**) Representative CD spectra comparing the secondary structure of HSA (control, dashed black), and HSA released from 1% alginate (black), 0.5% chitosan (red), 1% alginate|0.5% chitosan (green), and 2% alginate|1% chitosan (blue) after 3 days; and (**b**) specific activities of HRP released from 1% alginate (white bars), 0.5% chitosan (grey bars), 1% alginate|0.5% chitosan (horizontal striped bars), and 2% alginate|1% chitosan (vertical striped bars) after 3 days. Data shown are the average and standard error of triplicate measurements.

Protein encapsulation in hydrogels and other polymers has been used to protect and even increase their function and activity to enable various applications in biotechnology, including sensing [66,67,68,69]. To confirm if these benefits may also apply to therapeutic delivery, i.e., to facilitate the release of functional and active proteins, we used circular dichroism (CD) spectroscopy to compare the secondary structures of HSA prior to encapsulation (control) and HSA released from alginate and chitosan single- and double-network hydrogels. No major differences were observed among the CD spectra of the different samples (Figure 4a), thus indicating that the primarily α-helical secondary structure of HSA remained essentially unchanged due to protein encapsulation and upon its release from the hydrogels. While the characterization of the structure of released proteins may have been supported using protein conformation studies that used Fourier-transform infrared spectroscopy [70,71], we elected to use enzyme activity studies as a further verification of the observed preservation of protein structure. We compared the specific activities of the model enzymes, horseradish peroxidase (HRP), and β-Galactosidase (β-gal) prior to encapsulation and after their release from representative single- and double-network hydrogels. This experiment confirmed the preservation of protein function during its encapsulation in the hydrogels used in the study, as well as in its post-release from them (Figure 4b).

## 3. Discussion

While several proteins have been shown to have high therapeutic value against a wide range of human diseases [72,73], strategies for the efficient delivery of proteins are needed before their potential as therapeutics can be fully realized [74,75,76]. Polymers, especially hydrogels, have been demonstrated as attractive choices for protein delivery because of their capacity to provide a compatible environment for biologically active agents in their water-swollen network [12,14,16,77]. Furthermore, recent developments in preparing stimulus-responsive hydrogels, multicomponent hydrogels, nanogels, and fibers have advanced the applicability of hydrogels for the delivery of proteins [78,79,80,81]. Specifically, there is a growing interest in the use of hydrogel-based fibers for therapeutic delivery, as well as in other related fields, including tissue engineering and wound dressings [27,80,82,83,84,85,86]. Reasons for this increased interest in hydrogel fibers for delivery applications include their ease of fabrication, various fabrication methods to choose from, and tunable properties [23,87]. Furthermore, researchers have reported several strategies to control protein release from hydrogel fibers, including varying the surface area to volume ratio (by changing the fiber diameter), modifying the surface chemistry, and post-processing the fabricated fibers [22,24,88,89]. As an orthogonal approach, release rates of macromolecules from hydrogels may also be modulated by engineering the polymer concentration and crosslinking density [5,29,39]. In this study, we used two naturally occurring biopolymers—alginate and chitosan—to study the effects of polymer and crosslinker densities on the rates of protein release. Both hydrogels have been demonstrated to be efficient as tunable platforms for the controlled and sustained release of therapeutic molecules [90,91,92,93]. In this study, we demonstrate a strong negative correlation between hydrogel and crosslinker concentrations for alginate and chitosan, as well as protein release rates. Consistent with previous studies, chitosan hydrogels (but not alginate hydrogels) exhibit a pH-dependent release of proteins [93,94,95]. Furthermore, the pH-dependent behavior extended to DN hydrogels prepared using alginate and chitosan; not surprisingly, the effect of pH on the release of proteins from chitosan was attenuated in the presence of alginate. More importantly, our experiments confirm the ability to control protein release rates by preparing DN hydrogels that incorporate different ratios of alginate and chitosan. Taken together, the aforementioned results indicate that we can combine various properties of hydrogels to control the rates of protein release. Finally, our results demonstrate the preservation of structure and function for the proteins released from both the single- and double-network hydrogels. In conclusion, our work demonstrates a clear value for incorporating polymer properties that contribute to hydrogel porosity, as well as response to stimuli, as orthogonal control parameters in the design and development of hydrogel micro- and nanofibers for therapeutic protein release.

## 4. Materials and Methods

### 4.1. Materials

Materials for preparation of the hydrogels, alginate (low viscosity, 4–12 cps for 1% aqueous solution), calcium chloride (CaCl_2,_, ≥93%, granular), chitosan (low viscosity, 20–300 cps for 1% solution in 1% acetic acid), and sodium tripolyphosphate (TPP, technical grade, 85%) were purchased from Sigma-Aldrich (Saint Louis, MO, USA). Tris-HCl buffer (pH 7.2) was purchased from Life Technologies (Carlsbad, CA, USA). Micro bicinchoninic acid (µBCA) protein assay kit was obtained from Thermo Fisher Scientific (Waltham, MA, USA). Human serum albumin (HSA), horseradish peroxidase (HRP), and β-galactosidase (β-gal) were obtained from Sigma-Aldrich (St. Louis, MO, USA), as well as the substrates for quantifying the enzyme activity: 2,2′-azinobis(3-ethylbenzthiazoline-6-sulphonate) (ABTS), hydrogen peroxide (H_2_O_2_), and o-nitrophenyl β-D-galactoside (ONPG).

### 4.2. Preparation of Hydrogel Samples for Protein Release Studies

Proteins were encapsulated in the hydrogels by extrusion method, using a syringe and 25G needle. For alginate samples, alginate and protein stocks were diluted to their desired concentrations in pH 7.2, 250 mM Tris–HCl buffer, followed by gentle mixing using micro-pipetting and adding dropwise into 100 mM CaCl_2_ solution under stirring. For chitosan samples, the hydrogel–protein mixtures were added dropwise into 2% *w*/*v* TPP under stirring. For double-network hydrogels composed of alginate and chitosan, the hydrogel–protein mixtures were first added dropwise into 100 mM CaCl_2_ solution (to crosslink alginate), followed by incubation in 2% *w*/*v* TPP (to crosslink chitosan) under stirring. The final protein concentration in the hydrogel–protein mixtures for all samples was ca. 200 μg/mL. Final alginate concentrations for the single-network hydrogels were 1%, 2%, and 3% *w*/*v*, and chitosan concentrations were 0.5 and 1% *w*/*v*, respectively. For the DN hydrogels, final alginate concentrations were 1% and 2% *w*/*v*, and chitosan concentrations were 0.5 and 1% *w*/*v*, respectively, and the alginate to chitosan ratios were 1:1 and 2:1.

### 4.3. Measurement of Protein Release Rates

Protein release rates were determined by incubating the hydrogel–protein beads in either pH 7.2, 100 mM Tris–HCl buffer, or pH 4.5, 100 mM Tris–HCl buffer (prepared by adding stock HCl to Tris–HCl buffer and checked by a pH meter), at 37 °C. At specific time intervals, a sub-aliquot was removed from the samples to measure the amount of protein released by the hydrogels. The concentrations of proteins released from the hydrogels were quantified using the µBCA assay, per the manufacturer’s instructions. Final absorbance was measured at 560 nm using a Tecan Infinite 200 PRO spectrophotometer (Durham, NC, USA). The experiments were performed in triplicate, and the average drug release rates were calculated as

$$Protein\;release,\%=\frac{Protein\;released\;from\;the\;hydrogel}{Total\;protein\;in\;the\;hydrogel}\times 100$$

### 4.4. Measurement of Swelling Rates

To measure the swelling properties of the hydrogels, the hydrogel beads containing no protein were prepared using the extrusion method as described above. Initial weight (W_0h_) was determined at time = 0 after removing excess buffer using a micropipette. The samples were then immersed in either pH 7.2 or pH 4.5, 100 mM Tris–HCl buffer at 37 °C. Their final weights (W_72h_) were recorded at time = 72 h after removing excess buffer using a micropipette. The experiments were performed in triplicate, and the average swelling ratios were calculated as

$$Swelling\;ratio,\%=\frac{W_{72h}-W_{0h}}{W_{0h}}\times 100$$

### 4.5. Measurement of Protein Structure

Protein structure, prior to encapsulation and post-release from the representative hydrogels after three days, was determined using circular dichroism (CD) spectroscopy, using an Olis Rapid-Scanning Monochromator (Athens, GA, USA). Scans were taken in a quartz cylindrical cuvette with a pathlength of 1 mm. The recorded wavelength range was 185–260 nm, with the number of increments set to 150. Final protein concentration for all of the samples was set to ca. 50 μg/mL (initial protein concentrations prior to dilution to 50 μg/mL were estimated using the µBCA assay, as described above in Section 2.3).

### 4.6. Measurement of Enzyme Activity

Enzymatic activities, prior to protein encapsulation and post-release from the representative hydrogels after three days, were determined by measuring the initial rates of formation of colorimetric products using a Tecan Infinite 200 PRO spectrophotometer (Durham, NC, USA). HRP oxidizes ABTS at the expense of H_2_O_2_ to form a soluble end product that can be spectrophotometrically monitored at 405 nm, and β-gal catalyzes the hydrolysis of ONPG to o-nitrophenol, whose absorbance can be measured at 420 nm. ABTS and H_2_O_2_ concentrations for the HRP assay were 100 µM and 40 µM, respectively, and ONPG concentration was 80 µM for the β-gal assay. Specific activities for the enzymes were calculated by dividing the initial rates by the enzyme concentrations. Concentrations of the enzymes released from the hydrogels were estimated using the µBCA assay, as described above in Section 2.3.

### 4.7. Statistical Analysis

Average and standard errors were calculated using Microsoft Excel (v. 16.54), and the standard error was presented in the form of error bars in the graphs.

## Data Availability

Data is contained within the article.
